# Supplementary material for: Fractional Factorial Design to Evaluate the Synthesis and Electrochemical Transfer Parameters of h-BN Coatings
Source: Nanomaterials (Basel). 2023 Nov 22;13(23):2992. doi: 10.3390/nano13232992 (PMC10708287; doi:10.3390/nano13232992)
Supplement: Supplementary file 1 [file nanomaterials-13-02992-s001.zip › nanomaterials-2658476-supplementary.pdf]

Supplementary Material

Fractional Factorial Design to Evaluate the Synthesis and Electrochemical Transfer Parameters of h-BN Coatings.

Helen Figueroa [1,2], Juliet Aristizábal [1,3], Elías Reinoso-Guerra [1,4], Bárbara Arce [1], María José Vargas-Straube [1], Dana Gentil [1], Cristian Ramírez [2], José Cordero [5], Nelson P. Barrera [5] and Carolina Parra [1,4]*

[1] Laboratorio de Nanobiomateriales, Departamento de Ingeniería Mecánica, Universidad Técnica Federico Santa María, Avenida España 1680, Valparaíso 2390123, Chile; helen.figueroa.14@sansano.usm.cl (H.F.);
juliet.aristizabal@usm.cl (J.A.); elias.reinoso.12@sansano.usm.cl(E.R.-G.);
barbara.arce.lopez@gmail.com (B.A.); maru.vs@gmail.com (M.J.V.-S.); dana.gentil@usm.cl (D.G.)

[2] Departamento de Ingeniería Química y Ambiental, Universidad Técnica Federico Santa María, Avenida España 1680, Valparaíso 2390123, Chile; cristian.ramirez@usm.cl (C.R.)

[3] Departamento de Física, Universidad Técnica Federico Santa María, Avenida España 1680, Valparaíso 2390123, Chile

[4] Centro Científico Tecnológico de Valparaíso, Universidad Técnica Federico Santa María, Calle General Bari 699, Valparaíso 2340000, Chile

[5] Facultad de Ciencias Biológicas, Pontificia Universidad Católica de Chile, Alameda 340, Santiago 7820436, Chile; jocorderf@gmail.com (J.C.); nbarrera@bio.puc.cl (N.P.B.)

* Correspondence: carolina.parra@usm.cl (C.P.)

Material and Methods

Figure S1 shows a diagram depicting the system specifically designed for the synthesis of hexagonal boron nitride (h-BN).

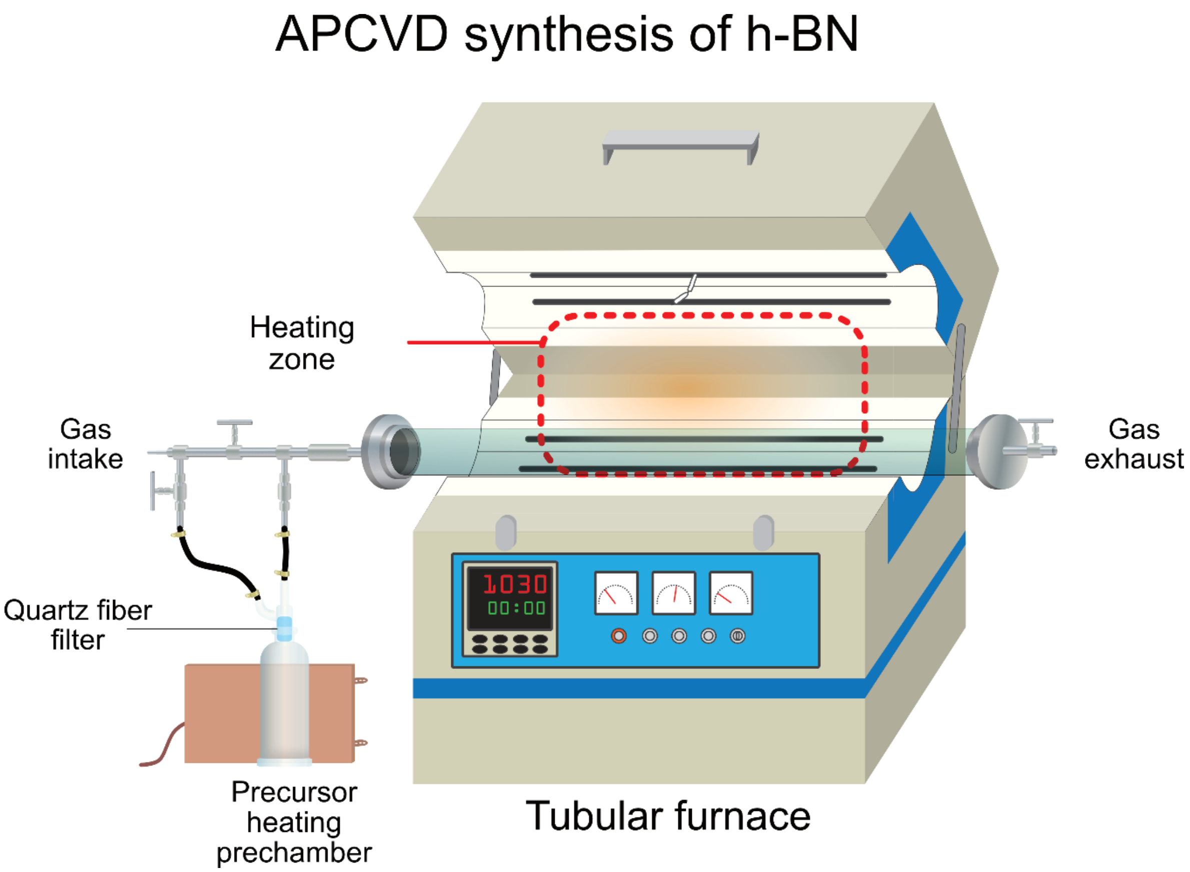

Figure S1. Schematic of the busy system to perform h-BN synthesis.

Characterization.

One of the elements that determines the quality of h-BN synthesis is the condition of the substrate. Depending on the preparation of the Cu substrate, the h-BN coating will exhibit variations in the coated area and domains with different grain sizes.

Prior to using copper as a substrate, it may have acquired an oxide layer on its surface, which can act as a self-cleaning substrate during synthesis [97]. This occurs because oxidation products in air (such as cuprous oxide or cupric oxide) thermally decompose at higher temperatures than metallic copper, releasing oxygen on the surface of the substrate[98]. This released oxygen reacts with the carryover gases and residual products

or precursors, causing the latter to promote the growth of grain-free h-BN on copper. This generally occurs because the oxygen released from some surface areas reacts with nitrogen and boron before reacting with the Cu surface (surface reaction).

To evaluate the influence of Cu substrate cleanliness on the h-BN coated area, we studied the effect of nitric acid pretreatment, used to remove or reduce the oxide layer prior to synthesis. This evaluation was carried out on samples synthesized using 11 [mg] of precursor with a synthesis time at 60 min. After synthesis the samples were heat treated in air to visualize areas with and without oxidation (without and with h-BN, respectively).

Figure S2a) and S2b) shows the areas where h-BN is deposited (bright orange) and where it is not, which are oxidized (reddish). In the pretreated samples (polished using HNO_3) there are larger areas coated with h-BN. Optical microscopy shows transition sectors between the areas with and without h-BN deposit. In Figure S2, f) an area of white color can be observed, that may be connected to the presence of oxygen that was not released from the synthesis during the annealing, which could oxidize the hexagonal boron nitride in the form of a white h-BNO compound [99].

In the sample without substrate pretreatment, it is observed that the h-BN deposition happens in a non-uniform way because, prior to the synthesis, the copper foil already presents oxidation, increasing the availability of oxygen on the surface that cannot be removed from the system during the annealing stage of the synthesis. In contrast, by occupying a pretreated substrate, the h-BN is deposited more homogeneously, leaving smaller uncoated areas, as seen in Figure S2a) c) and e), where a reddish hue was found. From these results we can understand that the HNO_3 pretreatment on the copper substrate decreases the initial oxidation on the surface of the Cu substrate, producing less oxygen release during the synthesis process and making the h-BN deposit manage to cover larger areas. Thus, subsequent analyses are carried out by performing this combined pretreatment of the copper substrate.

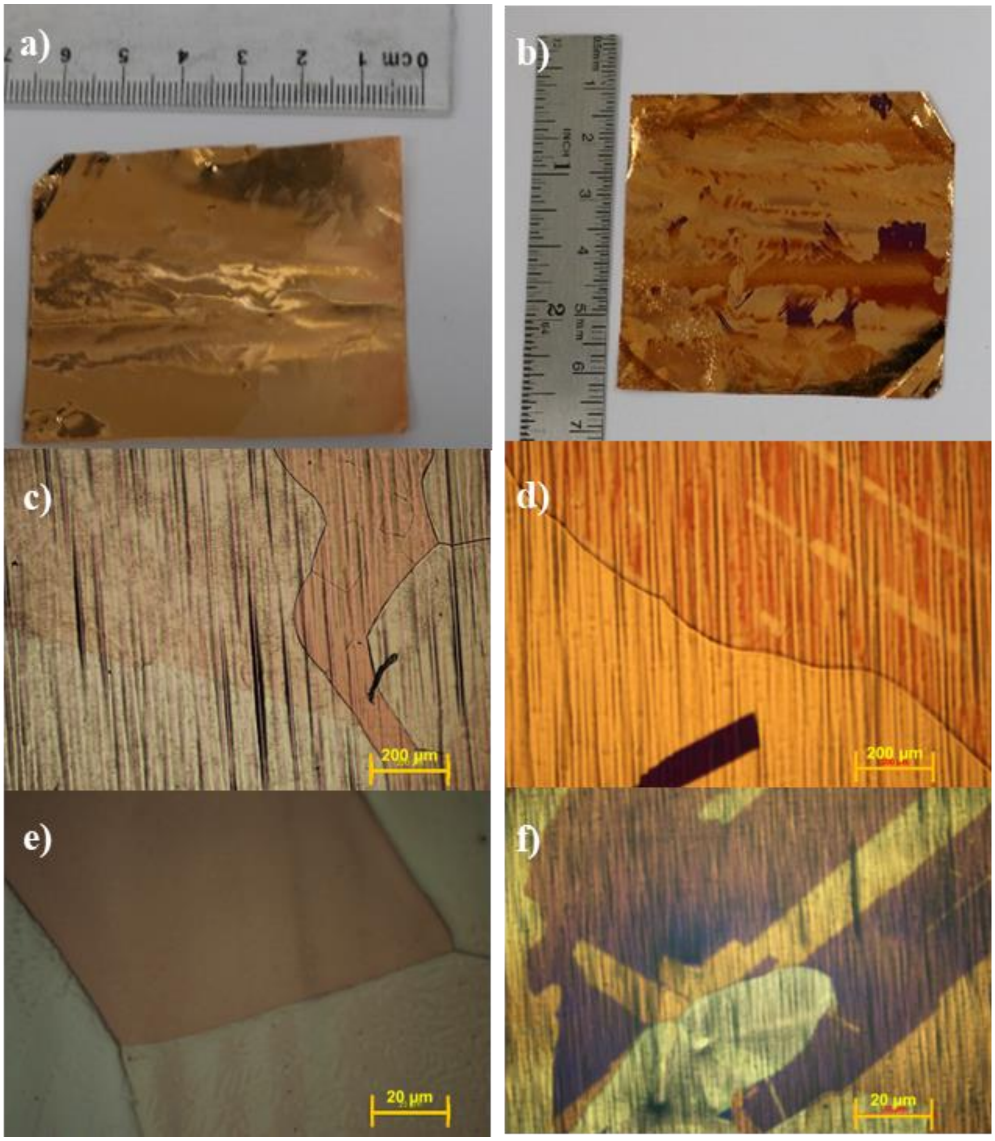

Figure S2. Resulting h-BN samples obtained using copper substrate treated with nitric acid and temperature. Photograph after synthesis using treated substrate a) and untreated Cu substrate b). Optical microscopy of h-BN of h-BN samples obtained using treated substrate c), e) and untreated Cu substrate d), f).

The surface composition of a Cu foil surface changes over time from just polycrystalline metallized copper (Cu) to a surface containing oxidation products such as cuprous oxide and cupric oxide, which form a layer that increases in thickness over time. This is observed in Figure S3 where a) polycrystalline copper without oxidation (pristine, recently purchased) and b) polycrystalline copper that presents oxidation due to the passage of time (oxidized, stored in air for three years), which present differences in color and brightness due to oxidation over time.

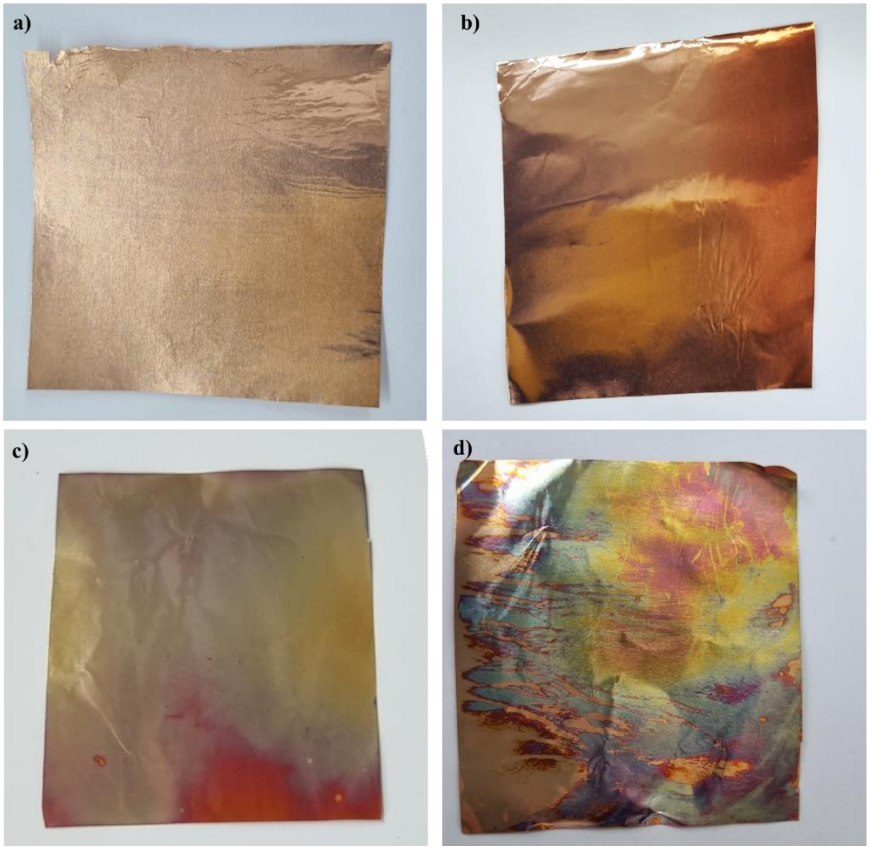

Figure S3: Copper sheet before pretreatment. a) Newly purchased copper (pristine) and b) copper oxidized in air for 3 years (oxidized). Copper foils heated up to 200° after nitric acid pretreatment c) Pristine and d) Oxidized copper.

Both foils were subjected to pretreatment with nitric acid (Figure S3) before heating in air. It is observed that the pristine copper foil (Figure S3, c) has a uniform surface appearance, while the oxidized copper foil (Figure S3, d) is non-uniformly oxidized.

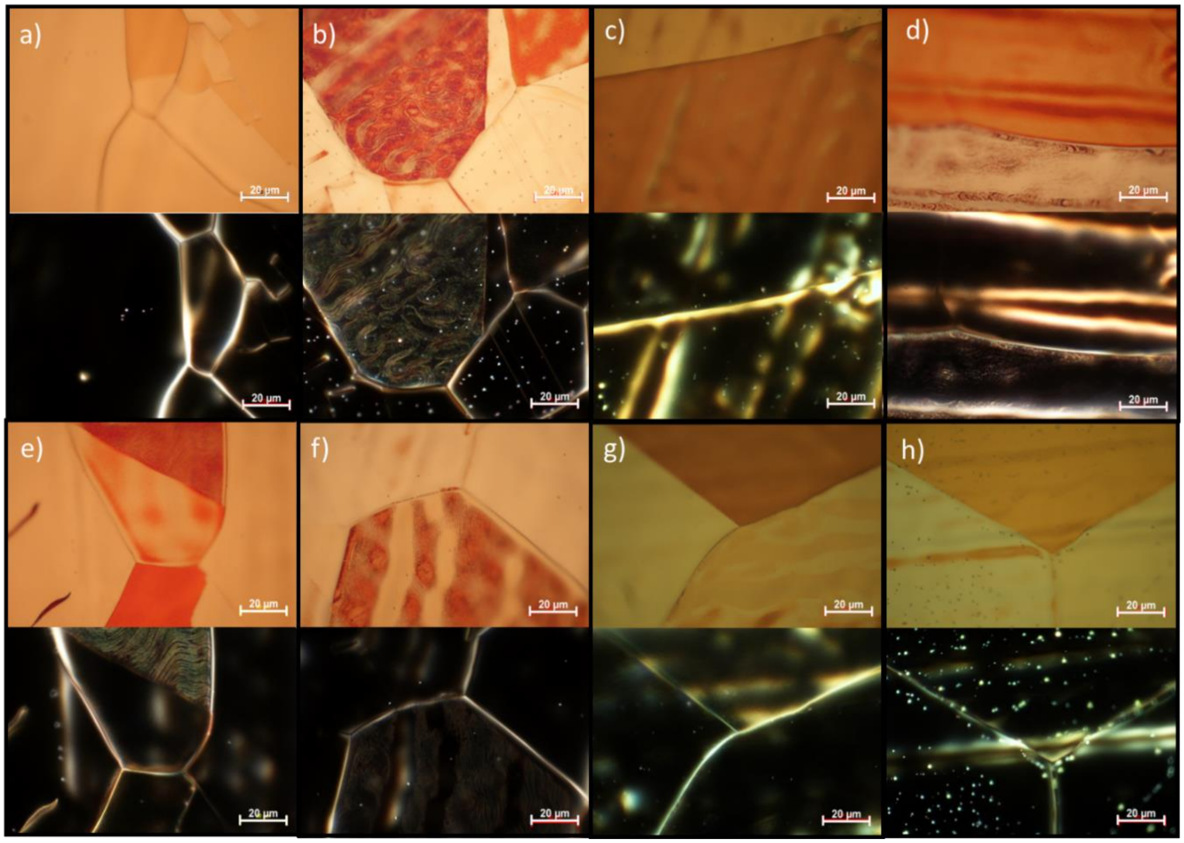

Figure S4. Optical microscopy of h-BN on copper for each experiment conditions.

The synthesized h-BN is intended to be used as a coating; through optical microscopy we can evaluate if a complete coverage of the copper surface was achieved the pretreatment strategy was successfully. In our case all the optical images show in general a complete coverage of the surface (Figure S4). In some cases, darker parts are observed which could be related to the presence of higher number of h-BN layers. In all cases the grain boundaries are well differentiated and are larger than 50μm. In the case of samples S_2 and S_8 there are luminous points that can be attributed to the formation of cubic boron nitride.

Table S1. p-value for each parameter for its determination of significance in the RMS roughness model

Source	p-value
Model	0,000
Lineal	0,000
A	0,005
B	0,073
C	0,000
D	0,800
E	0,000
2-term interactions	0,008
A*B	0,002
A*E	0,776

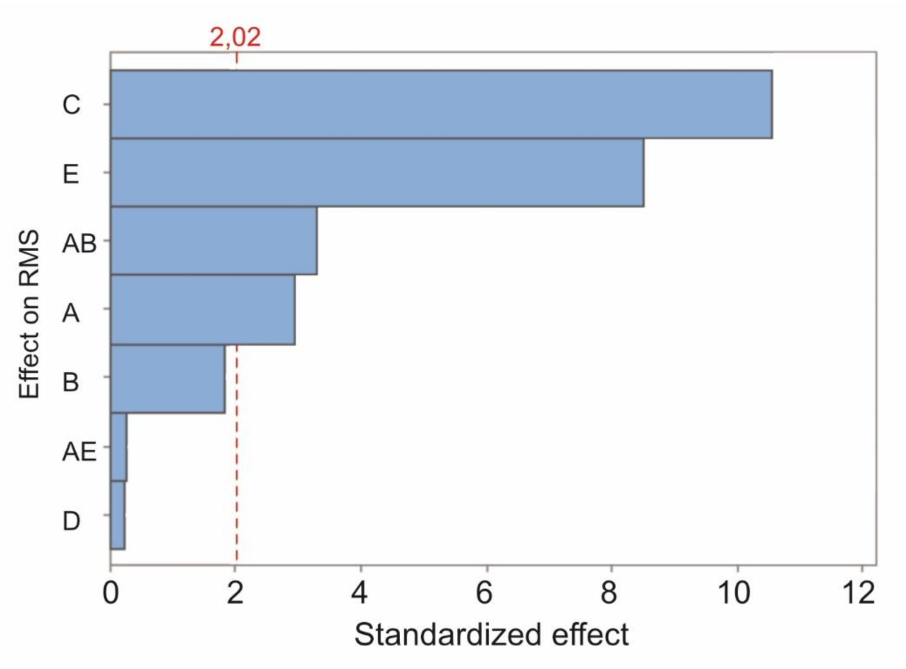

Figure S5. Pareto diagram of standardized effects for RMS roughness response (α=0.05).

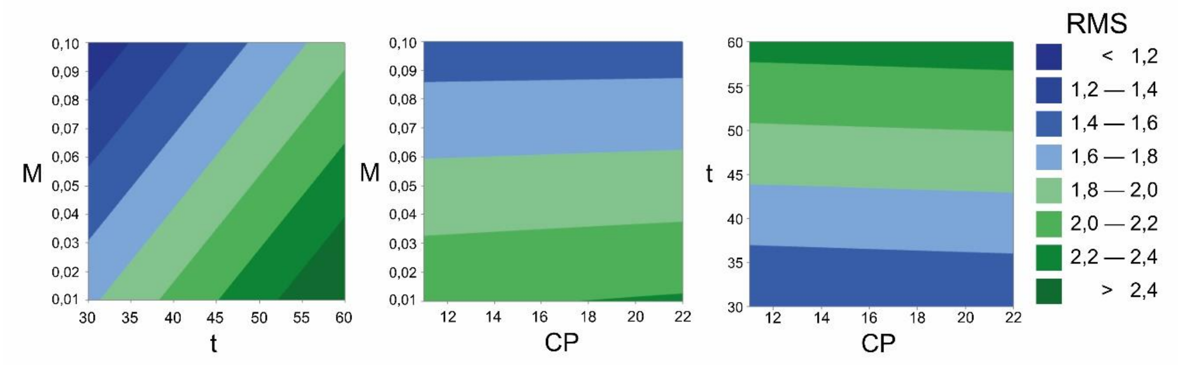

Figure S6. Contour plots between the relevant parameters related to the roughness of h-BN samples.

Table S2. p-value for each parameter for its determination of significance in the contact angle model

Source	p-value
Model	0,000
Lineal	0,000
A	0,007
B	0,000
C	0,000
D	0,384
E	0,078
2-term interactions	0,000
A*B	0,017
A*E	0,000

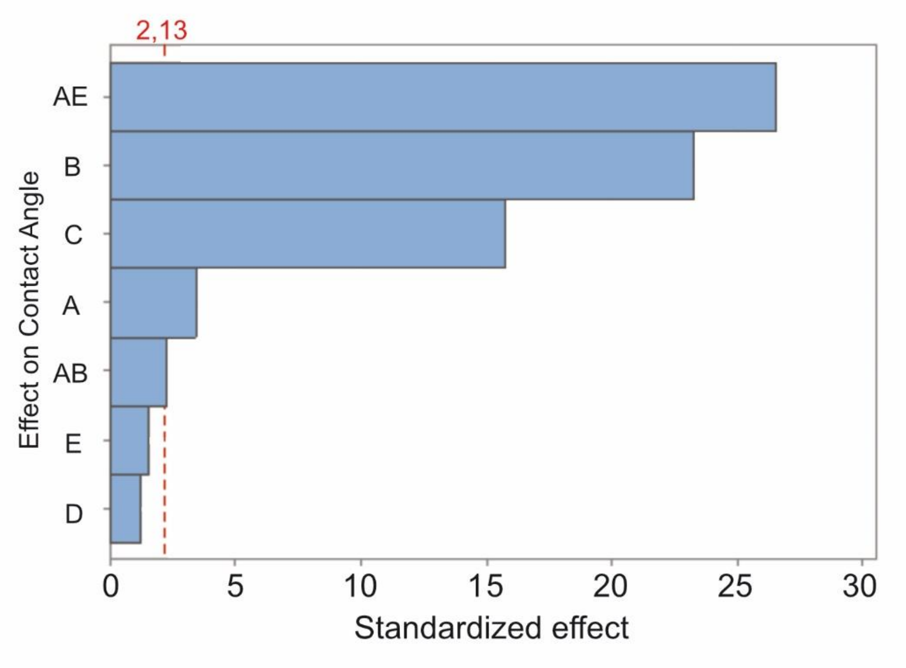

Figure S7. Pareto diagram of standardized effects for contact angle response (α=0.05).
